# In Vivo Antibacterial Efficacy of Nanopatterns on Titanium Implant Surface: A Systematic Review of the Literature

**DOI:** 10.3390/antibiotics10121524

**Published:** 2021-12-14

**Authors:** Yang Sun, Yang Yang, Weibo Jiang, Haotian Bai, He Liu, Jincheng Wang

**Affiliations:** 1 Orthopaedic Medical Center, The Second Hospital of Jilin University, Changchun 130041, China; sunyang@jlu.edu.cn (Y.S.); yangyang7019@mails.jlu.edu.cn (Y.Y.); weibo0515@163.com (W.J.); baihaotian@jlu.edu.cn (H.B.); heliu@jlu.edu.cn (H.L.); 2 Engineering Research Centre of Molecular Diagnosis and Cell Treatment for Metabolic Bone Disease, The Second Hospital of Jilin University, Changchun 130041, China

**Keywords:** titanium implant, nanopattern, surface morphology, antibacterial, biofilm formation

## Abstract

Background: Bionic surface nanopatterns of titanium (Ti) materials have excellent antibacterial effects in vitro for infection prevention. To date, there is a lack of knowledge about the in vivo bactericidal outcomes of the nanostructures on the Ti implant surfaces. Methods: A systematic review was performed using the PubMed, Embase, and Cochrane databases to better understand surface nanoscale patterns’ in vivo antibacterial efficacy. The inclusion criteria were preclinical studies (in vivo) reporting the antibacterial activity of nanopatterns on Ti implant surface. Ex vivo studies, studies not evaluating the antibacterial activity of nanopatterns or surfaces not modified with nanopatterns were excluded. Results: A total of five peer-reviewed articles met the inclusion criteria. The included studies suggest that the in vivo antibacterial efficacy of the nanopatterns on Ti implants’ surfaces seems poor. Conclusions: Given the small number of literature results, the variability in experimental designs, and the lack of reporting across studies, concluding the in vivo antibacterial effectiveness of nanopatterns on Ti substrates’ surfaces remains a big challenge. Surface coatings using metallic or antibiotic elements are still practical approaches for this purpose. High-quality preclinical data are still needed to investigate the in vivo antibacterial effects of the nanopatterns on the implant surface.

## 1. Introduction

In the past few decades, with the rapid development of material science and biomedical technology, biomedical metal materials have been widely used in the manufacture of artificial joints, bone trauma fixation, and other osseous tissue replacement or repair medical devices, where titanium (Ti) has become the preferred material due to its similarity with the biomechanical characteristics of human bones and the excellent biocompatibility [1,2]. While many researchers focus on improving the osseointegration ability of Ti implants, the occurrence of postoperative infection, as one of the most severe complications after implantation, cannot be overlooked [3,4]. The infection not only leads to the failure of the implant and the surgery but also increases the patients’ recovery period and makes an economic burden on both patients and the medical system [5]. Although surgical techniques and concepts of sterility have improved in recent years, the average postoperative infection rate is still as high as 2–5% [3,6,7].

The use of antibiotics is a common and effective way to control this issue, but the reason why implant-related infections are challenging to treat lies in the formation of bacterial biofilm on the implant surface. This structure is mainly composed of polysaccharide polymers secreted by bacteria, which can protect the bacteria and resist various antibiotics from outside the biofilm [8]. In addition, the system-administered anti-infection method can also result in a low concentration of drugs in the surgical area due to scars or fibrosis of the surrounding tissues, and thus affects the antibacterial efficiency [5,9,10]. Meanwhile, the extensive use of antibiotics brings the problem of microbial resistance. Surface modifications such as the antibiotic or metallic coating on the implant surface provides more peri-implant antibacterial activity than traditional methods, thus showing an excellent antibacterial effect [11]. However, the antibacterial ability of coating will gradually weaken with the release of antibacterial substances. Moreover, the antibacterial metal ions released from coatings have intrinsic cytotoxicity and may affect the osseointegration performance of Ti implants [12,13].

It has been shown that modifying the surface morphology of Ti materials without adding other chemical reagents can achieve long-term antibacterial effects and inhibit biofilm formation in vitro [14,15,16]. The presence of nano-protrusions on the bionic nanopatterns leads to the destruction and death of the microbes through direct contact and stress concentration [5,17]. The development of surface nanopatterns with efficient antibacterial properties may enable the better clinical application of Ti-related medical materials and address the bacterial resistance problem caused by antibiotic abuse [5]. However, the interactions between nanopatterns and bacteria can be multifaceted, and the antibacterial efficiency in vivo and the role of various factors in regulating bactericidal behavior of the nanopatterns on the Ti implant surface remain unclear [18,19].

This article summarizes and analyzes the preclinical studies of the nanoscale patterns’ antibacterial behaviors on the Ti implant surface to draw a clearer view of the in vivo antibacterial efficacy of bionic nanoscale patterns for the future Ti implant surface bactericidal modification research and applications.

## 2. Results

### 2.1. Identification and Selection of Studies

**Figure 1:**
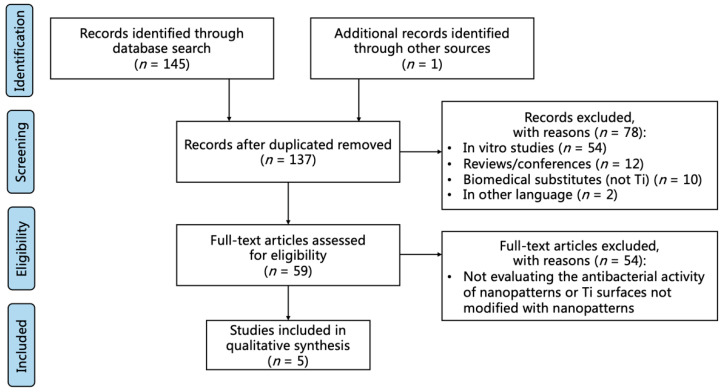
PRISMA (Preferred Reporting Items for Systematic Reviews and Meta-Analyses) diagram including study algorithm.

Electronic database searches identified 145 articles (Figure 1). After titles and abstracts were screened for relevance, 78 articles were deemed irrelevant per the inclusion and exclusion criteria. Of the 59 full texts of the in vivo animal studies assessed for eligibility, 5 were selected and reviewed after applying the inclusion and exclusion criteria. The 54 excluded articles did not evaluate the antibacterial activity of nanopatterns or did not modify Ti surfaces with nanopatterns. Meta-analysis was not conducted due to the scarcity and heterogeneity of the studies.

### 2.2. Study Characteristics

Table 1 and Table 2 provide a general description of the characteristics of the included studies. The five studies used three experimental models (rabbit [20], rat [21,22,23], and mice [24]). Sample sizes varied between 20 [22] and 45 animals [23]. All the studies performed the evaluation of the in vivo antibacterial activity of nanopatterns themselves on the surfaces of the Ti implants. Among these, one study focused on evaluating the antibacterial efficacy of nanopatterns alone on the implant surface [24]. Another evaluated the antibacterial effect of Ag and polydopamine as additional agents to the nanorods (NRDs) on the implant surface [22]. Two studies evaluated the antibacterial effect of metallic agents (Ag and Mg, respectively) as additional modifications on the NTs and NRDs, respectively, on the implant surface [22,23]. One study evaluated the antibacterial effect of fluorine (F)-incorporated NRDs on the implant surface [20]. The monitoring period ranged from 2 days [24] to 8 weeks [20]. All the studies assessed the antibacterial activity of nanopatterns alone on the surfaces. Only one study reported excellent antibacterial efficacy of nanopatterns alone on the implant surface [24]. The most frequently used shape of the implant was a cylinder, and the most used measurement method for in vivo antibacterial efficiency evaluation was bacterial culture and histopathological analysis.

### 2.3. Risk of Bias and Quality Assessment of the Studies

**Figure 2:**
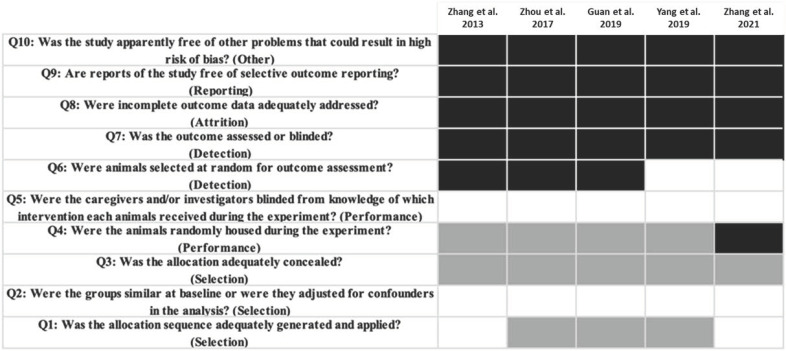
Risk of bias assessment results based on SYRCLE’s (Systematic Review Centre for Laboratory Animal Experimentation) risk of bias tool. White indicates low risk; black high risk; and gray unclear risk [20,21,22,23,24].

Assessment of the risk of bias according to SYRCLE (Systematic Review Center for Laboratory Animal Experimentation) guidelines was performed [25]. The included studies presented heterogeneous levels of risk of bias (Figure 2). The evaluation and scoring of the quality of the studies according to ARRIVE (Animal Research: Reporting of In Vivo Experiments) criteria [26,27] (Table S1) yielded an average score of 17.3. None of the studies reported item 19 (Replace, Reduce and Refine) or item 20 (Adverse events) in the abstract. Only one of the studies reported item 22 (Generalization/Applicability) [22] and item 5 (Reasons for animal models) [20]. One of the studies failed to report item 13 (Assignment of animals to experimental groups) [20].

## 3. Discussion

Modifying the surface nanopattern of materials to achieve antibacterial properties has attracted much attention in the past decade [14,15,16]. It has been shown that the surface morphology of insect wings such as dragonflies and cicadas has excellent antibacterial and antifungal properties [17,28,29]. With the presence of physical nano-protrusions on the surface of insect wings, the antibacterial properties may be attributable to the fact that when microbial cells encounter the surface protrusions, they increase the stress and deformation of the microbial cell membrane structure, leading to their destruction and ultimately leading to cell dissolution and deaths [5,17]. Investigating the surface nanostructure of insect wings and preparing bionic nanopatterns on Ti-based materials according to it has emerged as new ideas for preparing modern antibacterial implants. The modification of the surface morphology of Ti implant to obtain or improve antibacterial ability without adding other chemical reagents, such as silver (Ag) or antibiotics, has been widely reported in ex vivo studies [5,15].

However, the in vivo experimental studies that we retrieved and reviewed did not provide factual data to support this view. Only one study provided evidence that the surface nanostructure on Ti implant has anti-infective effects in vivo [24]. The remaining four articles either indicated that the nanopatterns did not exhibit antibacterial activity in vivo at all [20,22] or only showed limited antibacterial efficacy [21,23]. Moreover, the in vivo antibacterial activity mechanisms of the Ti implant surface morphology seem more complex than just physical puncturing. By modification of the surface morphology on Ti foil using TiO_2_ NRDs, Zhang et al. reported that the NRD arrays under irradiation with 808 near-infrared (NIR) light produced excellent antibacterial activity against *Staphylococcus aureus* in vivo through the combined actions of hyperthermia, reactive oxygen species (ROS), and puncturing effects, and could eradicate the attached biofilms on the implant surface in a Kumming mice model [24].

The effect of inhibiting the formation of biofilms is generally considered relevant to the anti-adhesion properties of the surface morphology [30,31,32]. Compared with the patterned surfaces, bacteria were reported more likely to adhere to smoother surfaces [32,33,34,35]. In the study reported by Guan et al., the bacterial coverage on the TiO_2_ NRDs samples was significantly lower than the bacterial coverage on the pure Ti group that formed a typical biofilm after 48 h ex vivo. The authors attributed this to the anti-adhesion effect of the topography on the implant. However, their in vivo experiment results using a Sprague Dawley rat model did not show the antibacterial activity of the TiO_2_ NRDs, and there was no difference in the infection rate between the TiO_2_ NRDs and pure Ti groups [22].

Antibacterial agents can be added to the surface of Ti materials to obtain/improve bactericidal properties [36,37,38]. Most of the studies in this regard were focused on the preparation of antibacterial metal nanoparticles (NPs) fixed on the surface of the Ti substrate by using a carrier or coated on the surface to achieve strong antibacterial ability, good biocompatibility, and stability [36,39,40]. The common metal particles selected for this purpose are Ag, Cu, zinc (Zn), magnesium (Mg), etc., which present antibacterial activities by generating ROS, destroying the structure of bacterial membranes, or regulating the signal transduction pathway of bacteria [41,42,43,44]. Among these, the advantages of a broad spectrum of antibacterial activity make Ag the most studied and widely used metal-based antibacterial agent on Ti substrates [45,46,47]. Ag NPs have been proven to have a good killing effect on both Gram-positive cocci (e.g., *S. aureus*) and Gram-negative bacilli (e.g., *Escherichia coli*) [48,49,50]. In the study reported by Guan et al., a novel surface strategy involving the formation of polydopamine (PDA) and Ag nanoparticle-loaded TiO_2_ NRDs coatings on Ti alloy was developed. In vitro antibacterial experiments showed that, compared to the pure Ti group, the Ag-TiO_2_@PDA NRDs coating group had adequate antibacterial effects at 7 and 14 days, according to the bacterial counting results. The efficacies were 88.6 ± 1.5% and 80.1 ± 1.1%, respectively, against methicillin-resistant *S. aureus*, and 91.3 ± 0.5% and 86.2 ± 2.6% against *E. coli*. Nevertheless, their TiO_2_ NRDs group also showed a 22.3 ± 3.9% antibacterial efficacy for methicillin-resistant *S. aureus* (MRSA) and 25.4 ± 12.3% for *E. coli*. During the in vivo experiments, the materials were implanted into the tibia of a MRSA infected Sprague Dawley rat model. After four weeks, the results of X-ray, micro-CT, and histopathological analysis showed that MRSA could be killed by Ag+, confirming that the Ag-TiO_2_@PDA NRDs coating also had good antibacterial activity in vivo. However, same as the pure Ti group, TiO_2_ NRDs on the surface did not show antibacterial activity in vivo [22].

Although the metallic agents can serve as excellent antibacterial elements on the Ti implant surface, the biggest challenge lies in enabling the stable release of such agents at a suitable concentration on the surface of the implants [51,52]. Metal ions released by the coatings are highly mobile and cytotoxic, and their entrance into living cells with high concentrations can kill healthy cells [53]. One solution to this problem is to create nanotube (NT) patterns on the surface of the substrate and load them into the structures for a controlled releasing and long-term antibacterial effect [47,54]. In the study performed by Yang et al., Mg-incorporated NT-modified Ti implants (NT-Mg) were designed and tested to measure the antimicrobial properties. The results demonstrated that NT-Mg implants maintained continuous and reliable release of Mg ion from the NTs, producing long-lasting antimicrobial activity both in vitro and in vivo. The nanotubular structure and alkaline microenvironment during degradation were the two main reasons responsible for the antimicrobial properties of NT-Mg. However, although the nanotubular structure itself exhibited slight anti-infection potential in vivo, the nanotubular structure alone could not combat such a severe implant-related bone infection [23].

In recent years, to avoid the cytotoxicity of the metal ion as the antibacterial agent, studies of non-metallic elements serving as the bactericidal agent of the surface of Ti implants have emerged [20,55,56,57]. It has been reported that fluorine (F)-doped nanopatterns on Ti material surfaces have excellent antibacterial ability against numerous bacteria in vitro and good cytocompatibility and osteoblastic activity [20,55,58,59]. In the study reported by Zhou et al., F-doped Sr_1_-HA (strontium containing hydroxyapatite) NRDs on microporous TiO_2_ implant led to the significantly improved antibacterial activities in a *S. aureus* infected New Zealand rabbit model at 8 weeks according to the bacteria counting results, and the effect was related to the incorporated F dose. However, the average CFU counting results exhibited the Sr_1_-HA NRDs on microporous TiO_2_ did not possess antibacterial activity against *S. aureus* in vivo at 8 weeks [20].

In addition to the inorganic antibacterial agents coating strategies, the antibiotic coating can also be added to the Ti implant surface to achieve antibacterial effects [60,61,62,63]. Under ideal conditions, the antibiotics loaded on the surface should release in a controlled path and speed, reach the effective drug concentration, and maintain a long sufficient sterilization time, reducing the risk of bacterial resistance problems caused by antibiotic abuse [64,65,66]. Considering that both aerobic and anaerobic organisms can cause bone infections and the high frequency of polymicrobial infections [67,68,69], broad-spectrum antibiotics such as rifampicin, gentamicin, vancomycin, etc., are recommended as the loaded agents [70,71]. Under ideal conditions, the antibiotics released by the prepared nanomaterials should reach the effective drug concentration and maintain a long sufficient sterilization time [64,72]. Zhang et al. compared the antibacterial efficacy of TiO_2_ NTs loaded with vancomycin (NT-V) with those of the NTs and commercially pure (Cp-Ti) groups in an *S. aureus* infected Sprague Dawley rat model. Compared with the other groups, NT-V showed an excellent antibacterial effect both in vitro and in vivo. Although the NTs reduced the surface bacterial adhesion in vitro, implant infection still developed in the in vivo experiments. The infection rate in the NT-V group was 0% at 30 days, while that of the NT and Cp-Ti groups reached 92% and 100%, respectively [21]. Although *S. aureus* is the primary pathogen responsible for bone infections [73,74,75], the evaluation of the in vivo anti-*Staphylococcus* activity alone further undermined the clinical translation value of the studies retrieved.

This systematic review has several limitations. Although a systematic literature search was performed and no similar reviews were identified, the registration of this systematic review was not completed before the data extraction was finished, and the limited number of studies and heterogeneity in reporting and experimental designs may influence outcomes, hinder result comparison, and preclude meta-analysis. Additionally, the findings of this review are supported by basic science studies (level 3). No studies of level 2 evidence or higher were identified. Finally, it is possible all relevant articles were not identified with our search criteria.

## 4. Materials and Methods

### 4.1. Systematic Literature Search

A comprehensive systematic literature search was conducted to answer the question, “Is the in vivo antibacterial efficacy of Ti implant enhanced by surface nanomorphology modification alone?” following the PRISMA (Preferred Reporting Items for Systematic Reviews and Meta-Analyses) guidelines [76]. The systematic search was conducted in August 2021 using three electronic databases: PubMed, Embase, and Cochrane. A general search was conducted using the following terms: (“nano”(All Fields) OR “nano-scale”(All Fields) OR “nanopattern”(All Fields) OR “nanomorphology”(All Fields) OR “nanostructure”(All Fields)) AND (“surface”(All Fields) OR “surfaces”(All Fields) OR “surfacing”(All Fields)) AND (“antimicrobial”(All Fields) OR “antibacterial”(All Fields)) AND (“titanium”(MeSH Terms) OR “titanium”(All Fields)) AND (“implantation”(All Fields) OR “implant”(All Fields) OR “implants”(All Fields)) AND “animals”(MeSH Terms).

The results were filtered to English and full-text articles. References from the articles were reviewed to confirm the completeness of the identified literature.

### 4.2. Exclusion and Inclusion Criteria

In vivo peer-reviewed studies evaluating the antibacterial efficacy of nanopatterns on Ti implant surfaces were included in this systematic review. Exclusion criteria for all studies were as follows: articles not written in English; review and expert opinion articles, conference proceedings, and presentations; ex vivo studies; studies that did not evaluate the antibacterial activity of nanopatterns or did not modify Ti surfaces with nanopatterns (Table S2). One author (Y.S.) performed the literature search, and two authors (Y.S., Y.Y.) independently reviewed the search results. Titles and abstracts were reviewed for all search results. Full-text articles were obtained to determine studies that met inclusion and exclusion criteria. If disagreement occurred, the senior author (J.W.) was consulted.

## 5. Conclusions

In contrast with the widely reported excellent in vitro antibacterial effectiveness, the in vivo antibacterial efficacy of the nanopatterns on Ti implants’ surfaces seems poor according to the preclinical studies we assessed. Moreover, given the small number of literature results, the variability in experimental designs, and the lack of reporting across studies, concluding the in vivo antibacterial effectiveness of the nanostructures on Ti substrates’ surfaces remains a big challenge. Surface modifications using metallic NPs or antibiotics coating are still practical approaches for achieving or improving the in vivo bactericidal activities. Despite our inability to ascertain the in vivo antibacterial efficacy of the surface nanopatterns, high-quality preclinical data are still needed to investigate the antibacterial effects of the nanopatterns on the implant surface and the mechanisms.

## Figures and Tables

**Table 1:** Study characteristics.

Studies	Animal Model (*n*)	Location of Implant Placement	Bacteria and Infection Set-Up	Follow-Up	In Vivo Antibacterial Efficacy Measures	In Vivo Antibacterial Efficacy Conclusions (Nanopatterns)
Zhang et al., 2013 [21]	Sprague Dawley rat (36)	Femoral intercondylar fossa	*S. aureus.* bacterial suspension (10^7^ CFU/100μL, 100 μL) was introduced into the femoral canal through the hole in the femoral intercondylar fossa.	30 days	Clinical assessment Bacterial Culture	Although better than the pure Ti, TiO_2_ NTs showed a poor antibacterial effect in vivo.
Zhou et al., 2017 [20]	New Zealand rabbit (24)	Left femur	PBS-diluted suspension of *S. aureus* (10^5^ CFU/mL, 20 μL) was injected into the medullary cavity of the femur.	8 weeks	Bacterial Culture CFU Counting	The Sr_1_-HA NRDs on microporous TiO_2_ showed no antibacterial activity in vivo.
Guan et al., 2019 [22]	Sprague Dawley rat (20)	Tibia plateau of the right knee	30 μL bacteria suspension (MRSA, 1.5 × 10^6^ CFU/mL) was injected into the exposed tibia hole.	4 weeks	X-ray Micro-CT Histopathological analysis	Same as the pure Ti group, TiO_2_ NRDs did not show antibacterial activity in vivo.
Yang et al., 2019 [23]	Sprague Dawley rat (45)	Femoral medullary cavity at the middle of intercondylar fossa	50 μL of PBS containing MRSA at a 1 × 10^6^ CFU/mL concentration was injected into the medullary cavity.	5 weeks	X-ray Micro-CT Histopathological analysis	The NT structure itself demonstrated limited antimicrobial activities in vivo.
Zhang et al., 2021 [24]	Kunming mice (24)	Subcutaneous tissue on the back and tibia	Antibacterial assay: samples were soaked in 50 μL of *S. aureus* (1 × 10^7^ CFU/mL) for 1 h and then implanted. Antibiofilm assay: samples were cultivated in 2 mL of the *S. aureus* solution (10^7^ CFU/ mL) to form biofilms.	2–14 days	Bacterial Culture Histopathological analysis	TiO_2_ nanostructures under the irradiation of 808 nm NIR light had an excellent anti-biofilm effect in vivo.

CFU: Colony-Forming Unit; HA: hydroxyapatite; *S. aureus: Staphylococcus aureus*; MRSA: methicillin-resistant *Staphylococcus aureus*; NRDs: nanorods; NTs: nanotubes; NIR: near-infrared; Sr: strontium; Ti: titanium.

**Table 2:** Implant characteristics and in vivo antibacterial activities (Outcomes).

Studies	Implants Number (n)	Implant Dimensions D(Ø) × L (mm)	Ti Implant Shape	Surface Nanopatterns	Nanopattern Dimensions D (Ø) × L (nm)	In Vivo Antibacterial Activities (Nanopatterns)
Zhang et al., 2013 [21]	36	1 (Ø) × L 20	Cylinder	TiO_2_ NTs	80 (Ø) × L 800	The infection rate was lower in the NT group compared to the Cp-Ti group (92% vs. 100%).
Zhou et al., 2017 [20]	120	2 (Ø) × L 10	Cylinder	Sr_1_-HA NRDs on microporous TiO_2_	NRDs Ø and interrod spacing: 70 ± 6 Pore Ø: 1000-3000	The average CFU counting results exhibited the Sr_1_-HA NRDs on microporous TiO_2_ did not possess antibacterial activity against *S. aureus* in vivo.
Guan et al., 2019 [22]	20	1 (Ø) × L 10	Cylinder	TiO_2_ NRDs	50–100 (Ø) × L 1000–2000	TiO_2_ NRDs showed no difference in the infection rate compared to the pure Ti group.
Yang et al., 2019 [23]	45	2 (Ø) × L 15	Cylinder	TiO_2_ NTs	80 (Ø)	The NT structure itself exhibited slight anti-infection potential in vivo, but the NTs structure alone could not combat such a severe implant-related bone infection.
Zhang et al., 2021 [24]	+	10 (length) × (wide) 5	Foil	TiO_2_ NRDs	Nanoleaf; NRDs: 40–50 (Ø) × L 1000	The TiO_2_ NRDs arrays under irradiation with 808 NIR light produced excellent antibacterial activity in vivo and could eradicate the attached biofilms on the implant surface.

CFU: Colony-Forming Unit; Cp-Ti: commercially pure titanium; HA: hydroxyapatite; NIR: near-infrared; PBS: phosphate buffered saline; Sr: strontium; *S. aureus: Staphylococcus aureus*; NRDs: nanorods; NTs: nanotubes; Ti: titanium.

## Data Availability

Not applicable.

## Supplementary Materials

The following are available online at https://www.mdpi.com/article/10.3390/antibiotics10121524/s1, Table S1: Checklist of ARRIVE criteria reported by the included studies, Table S2: Study inclusion and exclusion criteria.

## Abbreviations

Ag	silver
ARRIVE	Animal Research Reporting of In Vivo Experiments
CFU	Colony-Forming Unit
Cp-Ti	commercially pure titanium
Cu	copper
*E. coli*	*Escherichia coli*
F	fluorine
HA	hydroxyapatite
Mg	magnesium
MRSA	methicillin-resistant *Staphylococcus aureu**s*
NIR	near-infrared
NRD	nanorod
NT	nanotube
PBS	phosphate buffered saline
PDA	polydopamine
PRISMA	Preferred Reporting Items for Systematic Reviews and Meta-Analyses
ROS	reactive oxygen species
Sr	strontium
*S. aureus*	*Staphylococcus aureus*
SYRCLE	Systematic Review Center for Laboratory Animal Experimentation
Ti	titanium
Zn	zinc
