# Supplementary material for: In Vivo Antibacterial Efficacy of Nanopatterns on Titanium Implant Surface: A Systematic Review of the Literature
Source: Antibiotics (Basel). 2021 Dec 14;10(12):1524. doi: 10.3390/antibiotics10121524 (PMC8698789; doi:10.3390/antibiotics10121524)
Supplement: Supplementary file 1 [file antibiotics-10-01524-s001.zip › Table S1.pdf]

**Table S1. Checklist of ARRIVE criteria reported by the included studies**

| Items | Zhang et al. 2013 | Zhou et al. 2017 | Guan et al. 2019 | Yang et al. 2019 | Zhang et al. 2021 |
|---|---|---|---|---|---|
| 1. Title | 1 | 1 | 1 | 1 | 1 |
| Abstract | | | | | |
| 2. Species | 0 | 1 | 0 | 0 | 0 |
| 3. Key finding | 1 | 1 | 1 | 1 | 1 |
| Introduction | | | | | |
| 4. Background | 1 | 1 | 1 | 1 | 1 |
| 5. Reasons for animal models | 0 | 1 | 0 | 0 | 0 |
| 6. Objectives | 1 | 1 | 1 | 1 | 1 |
| Methods | | | | | |
| 7. Ethical statement | 1 | 1 | 1 | 1 | 1 |
| 8. Study design | 1 | 1 | 1 | 1 | 1 |
| 9. Experimental procedures | 1 | 1 | 1 | 1 | 1 |
| 10. Epermental animals | 1 | 1 | 1 | 1 | 1 |
| 11. Accommodation and handing of animals | 0 | 1 | 0 | 0 | 1 |
| 12. Sample size | 1 | 1 | 1 | 1 | 1 |
| 13. Assignment of animals to experimental groups | 1 | 0 | 1 | 1 | 1 |
| 14. Anesthesia | 1 | 1 | 1 | 1 | 1 |
| 15. Statistical methods | 1 | 1 | 1 | 1 | 1 |
| Results | | | | | |
| 16. Experimental results | 1 | 1 | 1 | 1 | 1 |
| 17. Results and estimation | 1 | 1 | 1 | 1 | 1 |
| Discussion | | | | | |
| 18. Interpretation and scientific implications | 1 | 1 | 1 | 1 | 1 |

| | | | | | |
|---|---|---|---|---|---|
| 19. Replace, Reduce and Refine | 0 | 0 | 0 | 0 | 0 |
| 20. Adverse events | 0 | 0 | 0 | 0 | 0 |
| 21. Study limitations | 1 | 0 | 1 | 0 | 0 |
| 22. Generalization/applicability | 0 | 0 | 1 | 0 | 0 |
| 23. Funding | 1 | 1 | 1 | 1 | 1 |
| TOTAL SCORE | 17 | 18 | 18 | 16 | 17 |
