# Supplementary material for: In Vivo Antibacterial Efficacy of Nanopatterns on Titanium Implant Surface: A Systematic Review of the Literature
Source: Antibiotics (Basel). 2021 Dec 14;10(12):1524. doi: 10.3390/antibiotics10121524 (PMC8698789; doi:10.3390/antibiotics10121524)
Supplement: Supplementary file 1 [file antibiotics-10-01524-s001.zip › Table S2.pdf]

Table S2. Study Inclusion and Exclusion Criteria

Type of Study	Inclusion Criteria
In vivo	Animal studies reporting the antibacterial activity of nanopatterns on Ti implant surface
	**Exclusion Criteria**
All	Ex vivo studies<br>Articles not written in English<br>Review and expert opinion articles, conference proceedings, presentations<br>Studies not evaluating the antibacterial activity of nanopatterns on Ti implant surface or not using Ti implant with nanopatterns on surface.
